# Molecular and Morphological Characterization of *Fasciola* spp. Isolated from Different Host Species in a Newly Emerging Focus of Human Fascioliasis in Iran

**DOI:** 10.1155/2014/405740

**Published:** 2014-06-11

**Authors:** Reza Shafiei, Bahador Sarkari, Seyed Mahmuod Sadjjadi, Gholam Reza Mowlavi, Abdolali Moshfe

**Affiliations:** ^1^Department of Parasitology and Mycology, School of Medicine, Shiraz University of Medical Sciences, P.O. Box 7134845794, Shiraz, Iran; ^2^Department of Parasitology and Mycology, School of Public Health, Tehran University of Medical Sciences, Tehran, Iran; ^3^Cellular and Molecular Research Center, Yasuj University of Medical Sciences, Yasuj, Iran

## Abstract

The current study aimed to find out the morphometric and genotypic divergences of the flukes isolated from different hosts in a newly emerging focus of human fascioliasis in Iran. Adult *Fasciola* spp. were collected from 34 cattle, 13 sheep, and 11 goats from Kohgiluyeh and Boyer-Ahmad province, southwest of Iran. Genomic DNA was extracted from the flukes and PCR-RFLP was used to characterize the isolates. The ITS1, ITS2, and mitochondrial genes (mtDNA) of NDI and COI from individual liver flukes were amplified and the amplicons were sequenced. Genetic variation within and between the species was evaluated by comparing the sequences. Moreover, morphometric characteristics of flukes were measured through a computer image analysis system. Based on RFLP profile, from the total of 58 isolates, 41 isolates (from cattle, sheep, and goat) were identified as *Fasciola hepatica*, while 17 isolates from cattle were identified as *Fasciola gigantica*. Comparison of the ITS1 and ITS2 sequences showed six and seven single-base substitutions, resulting in segregation of the specimens into two different genotypes. The sequences of COI markers showed seven DNA polymorphic sites for *F. hepatica* and 35 DNA polymorphic sites for *F. gigantica*. Morphological diversity of the two species was observed in linear, ratios, and areas measurements. The findings have implications for studying the population genetics, epidemiology, and control of the disease.

## 1. Introduction

Fascioliasis, caused by the liver flukes of the genus* Fasciola*, is one of the most important food- and water-borne parasitic zoonoses [1].* Fasciola hepatica* and* Fasciola gigantica* are two main species of* Fasciola* which infect both human and animals. While* F. gigantica* is occurring mainly in tropical and* F. hepatica* in temperate areas, both species overlap in subtropical zones [2].

The two species have been traditionally classified based on their morphological features, such as body length and width. Because of variations in size of these two species, the discrepancy of morphological features, and the presence of intermediate forms, it might be difficult to distinguish the two species, solely based on these characters [3].

Recent molecular studies demonstrated that the two species can be properly distinguished by DNA sequencing of ITS1 and ITS2 and also mitochondrial genes of NDI and COI [4–6].

Human and animal fascioliasis are a serious health and veterinary problem in Iran [7, 8]. Animal fascioliasis is quite common in grazing animals in most areas of the country and its prevalence reaches up to 50% in some provinces [8, 9]. During the past two decades, the disease emerged as a serious problem in the Northern Province of Gilan in Iran [7]. This province experienced two outbreaks of human fascioliasis in 1987, affecting more than 10,000 people, and, in 1997, affecting several thousands of people [9]. Moreover, cases of human fascioliasis have been reported from other provinces of Iran including Kohgiluyeh and Boyer-Ahmad in the southwest of the country [10]. In a recent study, a seroprevalence rate of 1.86% was reported for human fascioliasis in this province [10]. Study by Moshfe et al., about animal fascioliasis in this area, showed that 12.5% of cattle, 11.75% of sheep, and 7.16% of goats are infected by* Fasciola* spp. [11].

Several molecular studies have been conducted in north and south of Iran for genotype analysis of* Fasciola* spp. isolated from different host species [12–16]. However, such study has not been performed in Kohgiluyeh and Boyer-Ahmad province, as a new focus of human fascioliasis in Iran. In view of that, the objective of the present study was to characterize* Fasciola* samples isolated from different host animals in order to find out any morphometric and genotype differences within and between the isolates.

## 2. Material and Methods

### 2.1. Study Area

**Figure 1:**
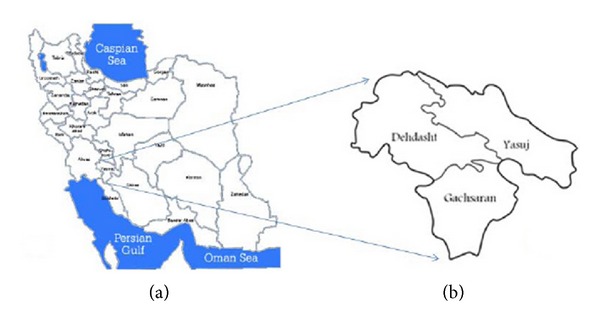
Map of Iran (a) and the Kohgiluyeh and Boyer-Ahmad province (b).

The Kohgiluyeh and Boyer-Ahmad province is located in southwest of Iran, with geographical coordinates between latitudes 30–9° to 31–27°N. and longitudes 49–55° to 51–42°E. (Figure 1). The province is characterized by a moderate and cool climate around its capital (Yasuj) in the east and temperate climate around the southwest of the province. The area is covered with oak trees and there are many valleys with rivers and waterfalls. Farming and stock breeding (cattle, sheep, and goat) dominated the lives of most of the people in this area. Moderate temperatures, rainfall during the year, and large pasture for ruminants provide suitable conditions for transmission and establishment of fascioliasis in this area.

### 2.2. Fasciola Samples

Adult Fasciola spp. were collected from 34 cattle, 13 sheep, and 11 goats at two slaughterhouses (Yasuj abattoir in the capital of the province and Gachsaran abattoir in the southwest of province) in Kohgiluyeh and Boyer-Ahmad province. Sources of the flukes were all from the same locality which was Kohgiluyeh and Boyer-Ahmad province in southwest of Iran. Individual flukes (one fluke from each liver) were washed extensively in PBS and fixed in 70% ethanol for extraction of genomic DNA.

For morphometric analysis, individual flukes were washed, three times, in PBS and fixed and stained in FAAL (formalin, azocarmine, alcohol, and lactic acid) solution followed by mounting with a medium containing poly vinyl alcohol [17].

### 2.3. Morphometric Measurement

The morphometric characteristics of the isolates were measured through a computer image analysis system (CIAS) based on the standardized measurements which are known to be suitable for the differentiation of both fasciolid species [18]. Olysia Software (Olysia zoom 3.2 Soft Imaging Systems 2003) compatible with Olympus Stereo Microscopes (SZX16) and digital camera (DP12) installed on a personal computer was employed to measure the morphometric criteria of adult* Fasciola*.

### 2.4. Statistical Analysis

Student's *t*-test was used to compare the mean of different variables between* F. hepatica* and* F. gigantica* and one-way analysis of variance (ANOVA) was used to determine whether there are any significant differences between the means of morphometric values in flukes isolated from different hosts.

### 2.5. DNA Extraction and Amplification

For extraction of genomic DNA, a portion of the apical and lateral zone of adult flukes, not including the reproductive organs, was removed and crushed. DNA from the crushed materials was extracted using phenol-chloroform method [15].

DNA fragments of each target gene were amplified by polymerase chain reaction [19]. The PCR reactions (25 *μ*L) were performed with 3 *μ*L of DNA solution, 1.25 units of* Taq* DNA polymerase (Cinnagen, Iran), 2.5 *μ*L of 10x PCR buffer, 2 mM of MgCl_2_, 50 pmol/25 mL reaction mixtures of both forward and reverse primers, 0.4 mM of dNTPs, and 15.5 *μ*L of distilled water. In this study we used the primers which have been reported by Itagaki et al. [19].  Table 1 shows the sequences of the primers.

PCR amplification was performed in Eppendorf Mastercycler Gradient thermocycler programmed for one cycle of 90 s at 94°C, 30 cycles of 90 s at 94°C, 90 s at 55°C, 120 s at 72°C, and a final extension of 72°C for 10 min followed by cooling at 4°C.

### 2.6. PCR-RFLP Analysis

A PCR-RFLP method was used to specifically distinguish* F. hepatica* from* F. gigantica* in ITS1 with* RsaI* enzyme [20] and in IST2 with* MspI* and* KpnI* [21].

### 2.7. DNA Sequencing and Phylogenetic Analysis

PCR products of ITS1, ITS2, COI, and NDI of six isolates and two samples from each host (cattle, sheep, and goat) were purified from the agarose gel, using PCR purification kit (Bioneer, Korea), and sequenced from both directions (Applied Biosystems, DNA Analyzers Sequencing, Bioneer, Korea, Sanger method), using the same primers which were used in the PCR.

The sequences were aligned and compared with those of existing sequences (six sequences for each gene, from the region, Asia, and also from other region, Africa) related to* Fasciola* spp. available in the GenBank, using the BLAST program of GenBank. Multiple alignment was performed with data related to* Fasciola* spp. from Iran and other countries deposited in GenBank, using BioEdit Sequence Alignment Editor version 7.1.3 software [22].

A maximum likelihood tree was constructed using the MEGA 5.0 software [4]. Bootstrap analyses (using 1,000 replicates) were carried out to determine the robustness of the finding [4].

## 3. Results

### 3.1. Morphometric Analysis

**Figure 2:**
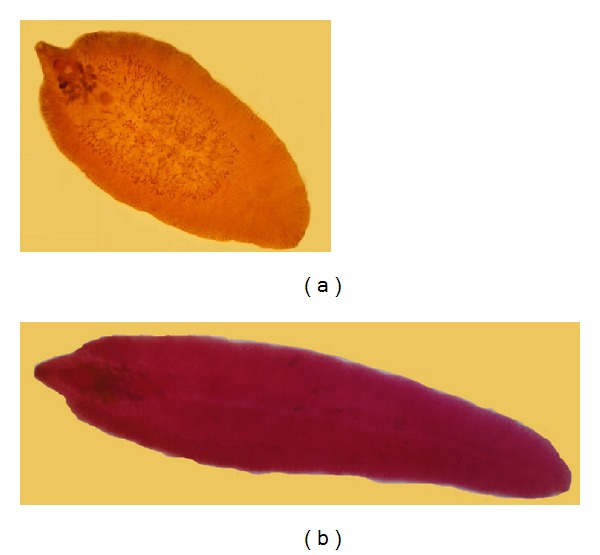
*Fasciola hepatica* (a) isolated from sheep and* Fasciola gigantica* (b) isolated from cattle.

Morphometric criteria taken up in this study consisted of 26 different parameters, which are known to be suitable for the differentiation of both* Fasciola* species, based on lineal biometric characters, areas, and ratios.  Figure 2 shows the overall feature of* F. hepatica* and* F. gigantica* isolated from sheep and cattle, respectively. The morphometric characteristics of isolated flukes from goat, sheep, and cattle are given in Table 2.

Morphological diversity in adult flukes of* F. hepatica* and* F. gigantica* was seen in few of characters including body length, body width, and body area. Analysis of morphometric criteria with *t*-test showed that the differences between the body length, body width and distance between the union of the vitelline glands and posterior end of the body in two species are significant (*P* < 0.05).

Analysis of morphometric features, using ANOVA, demonstrated that the differences in parameters including body length and width, body area, distance between the ventral sucker and union of the vitelline glands, distance between the ventral sucker and the posterior end of the body, testicular space length and width, maximum and minimum diameter of the ventral sucker, and ventral sucker area in* F. hepatica* from different hosts were statistically significant (*P* < 0.05) (Table 2).

Considering the morphometric characteristics of adult flukes with those of molecular findings, significant relationship was found between these features in* F. hepatica* and* F. gigantica* (*P* < 0.05).

### 3.2. PCR-RFLP Analysis

Based on RFLP pattern, from 58* Fasciola* isolates, 17 isolates (29.3%) from cattle had a RFLP pattern corresponding to* F. gigantica* and the rest of 41 samples (70.4%) from cattle sheep and goat had RFLP profile corresponding to* F. hepatica*. Molecular findings revealed that isolated flukes from sheep and goat were all* F. hepatica* whereas cattle were infected with either* F. hepatica* or* F. gigantica* (but not mixed infection).

### 3.3. Genotype Analysis Based on the ITS1 and ITS2 Ribosomal DNA

**Figure 3:**
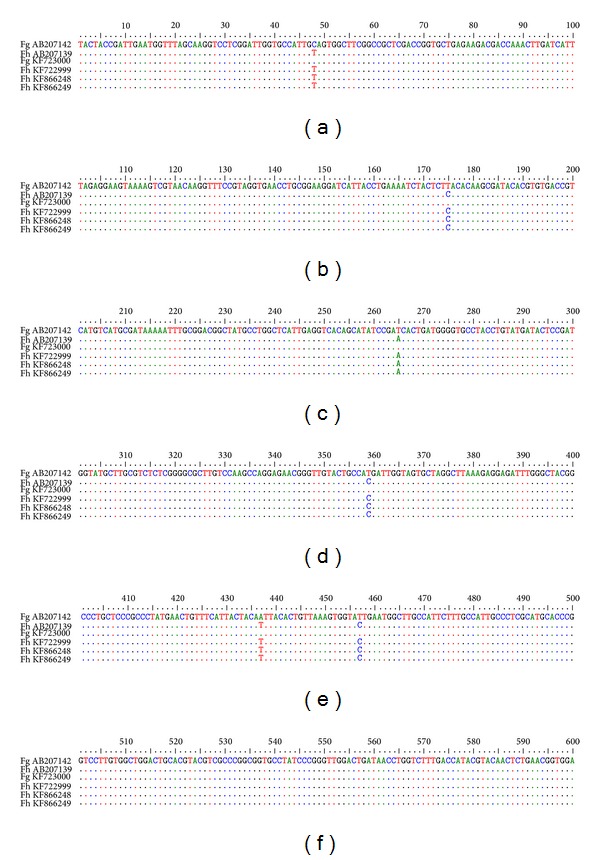
Alignment of ITS1 sequence of* F. hepatica* (AB207139) and* F. gigantica* (AB207142), with* F. gigantica* and* F. hepatica* of Kohgiluyeh and Boyer-Ahmad province, Iran.

**Figure 4:**
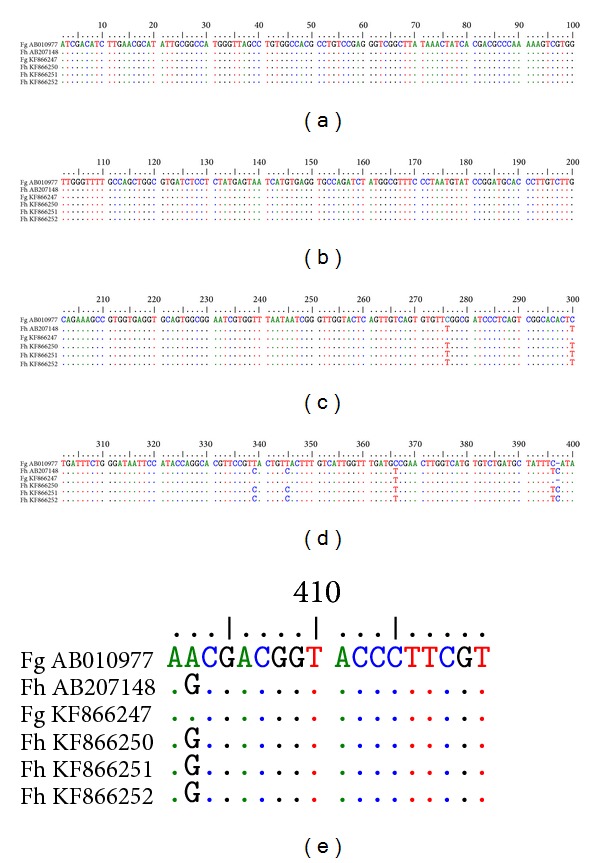
Alignment of ITS2 sequence of* F. gigantica* (AB010977) and* F. hepatica* (AB207148), with* F. gigantica* and* F. hepatica* of Kohgiluyeh and Boyer-Ahmad province, Iran.

Complete sequences of 600 bp ITS1 and 505 bp ITS2 of the flukes were aligned with those of available sequences in GenBank (Figures 3 and 4). Alignment of the sequences of ITS1 showed six DNA variable sites in which nucleotides at the position of 48, 175, 265, 359, 437, and 457 were single-base substituted resulting in segregation of the specimens into two different groups (genotypes). The main differences between* F. gigantica* and* F. hepatica* were the single-base substitution of C>T at nucleotide site of 48, T>C at the sites of 175, 359, and 457, T>A at the site of 265, and A>T at the site of 437.

Alignment of the sequences of ITS2 showed seven single-base substitutions at the position of 300, 339, 345, 367, 396, 397, and 402 resulting in segregation of the specimens into two different groups (genotypes). Sequences of ITS1 and ITS2 of* F. hepatica* and* F. gigantica* were deposited in GenBank (accession numbers: KF72299 to KF72300 and KF866247 to KF866252).

### 3.4. Genotype Analysis Based on the NDI and COI mDNA Markers

Mitochondrial NDI and COI gene markers were used to evaluate genetic diversity of flukes isolated from different hosts. Partial sequences (438 bp) of COI showed 42 variable sites and 6 haplotypes. Moreover, partial sequences of NDI (535 bp) showed 48 variable sites and yielded six haplotypes. Sequences of COI and NDI of the isolates were deposited in the GenBank (accession numbers: KF992216 to KF992227).

### 3.5. Phylogenetic Analysis

**Figure 5:**
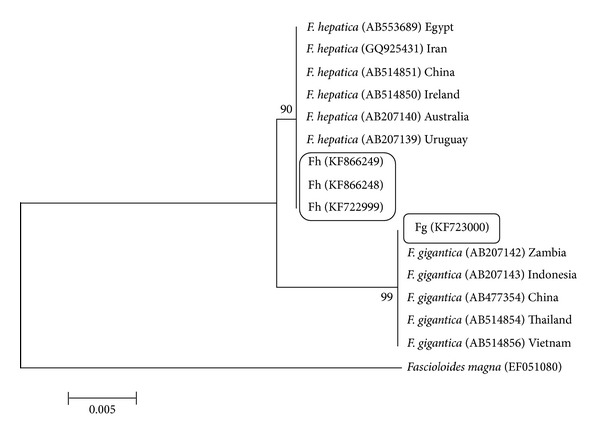
Phylogenetic relationship of ITS1 sequences of isolates of* Fasciola hepatica* and* Fasciola gigantica* from Iran using Maximum Likelihood method.* Fascioloides magna* (AN: EF051080) was used as the out group.

**Figure 6:**
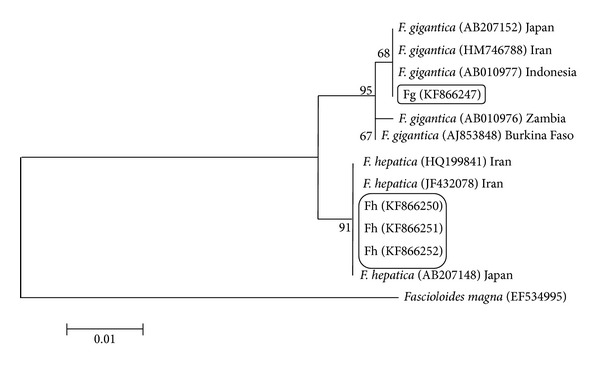
Phylogenetic relationship of ITS2 sequences of isolates of Fasciola hepatica and Fasciola gigantica from Iran using Maximum Likelihood method.* Fascioloides magna* (AN: EF534995) was used as the out group.

**Figure 7:**
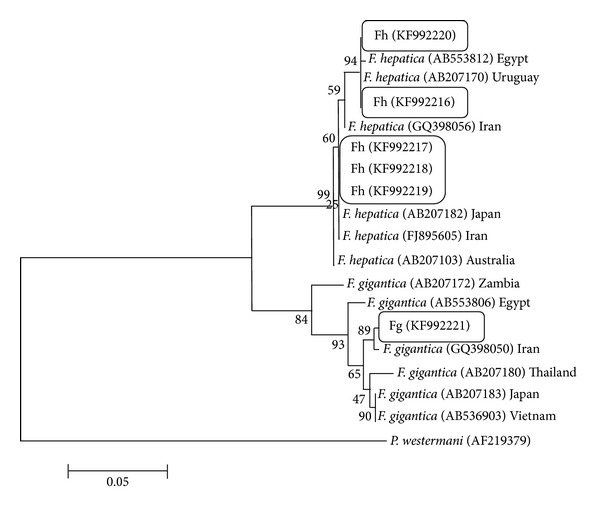
Phylogenetic relationship of COI sequences of isolates of* Fasciola hepatica* and* Fasciola gigantica* from Iran using Maximum Likelihood method.* Paragonimus westermani* (AN: AF219379) was used as the out group.

**Figure 8:**
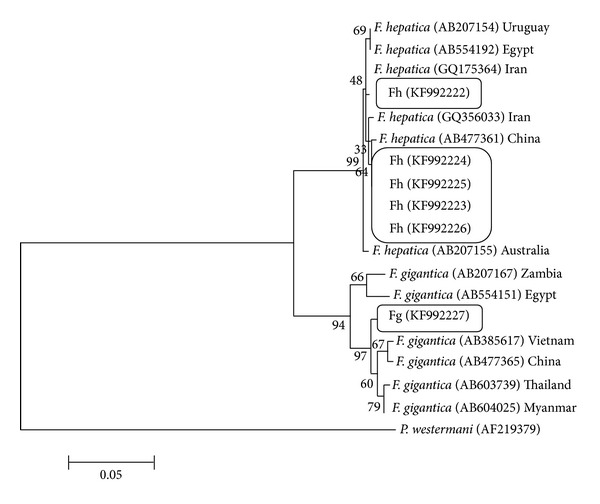
Phylogenetic relationship of NDI sequences of isolates of* Fasciola hepatica* and* Fasciola gigantica* from Iran using Maximum Likelihood method.* Paragonimus westermani* (AN: AF219379) was used as the out group.

For analysis of phylogenetic diversity of the flukes, phylogenetic trees were built, using ITS1, ITS2, NDI, and COI sequences of* F. gigantica* and* F. hepatica* from the present study along with other available sequences from the region, Asia, and also from another region, Africa (Figures 5, 6, 7, and 8).

In ITS1, the results showed that a close relationship is present between two* Fasciola* species of this study in comparison with isolates from other areas in the world. Considering the ITS2 sequences, all the sequences of* F. hepatica* sit in the same group but the values in the bootstrap test of phylogenetic accuracy indicated that the sequence of* F. gigantica* in this region, with some differences, sits in different branch. The* F. gigantica* of our study is similar to the* Fasciola* species of Zambia.

Sequence of COI of a goat in our study is similar to Uruguay isolate and one of the sheep samples is similar to Japanese isolates. Other sequences of* F. hepatica* are laid in the separate cluster. The only sample of* F. gigantica* which was collected from Gachsaran abattoir, south of the province, is more similar to strain of* F. gigantica* from Ahwaz which again is situated in the south of Iran.

## 4. Discussion

Fascioliasis is one of the most common helminthic infections of domestic livestock, in particular, in cattle, sheep, and, occasionally, in human in tropical and subtropical countries [1].

Findings of the current study show that adult flukes from goats, sheep, and cattle are significantly different in some of their allometries. Flukes from sheep and goat were somewhat larger than cattle. As pointed out by Valero et al., this might be due to faster growing of* F. hepatica*, which reaches a larger size in sheep and goat as compared to cattle [3]. Levels of host resistance and calcification in bile ducts of infected cattle have also been attributed to this difference in the size of adult fluke in different hosts [13, 23].

The study also demonstrated that morphometric indices can also be used for differential diagnosis of the two* Fasciola* species. The findings have implications for studying the population genetic, epidemiology, and control of the disease in the region.

Previous studies demonstrated that molecular phylogeny with help of nucleotides of mtDNA, including NDI and COI, and rDNA, including ITS1 and ITS2 genes, can be effectively used for proper differentiation of fasciolids as well as elucidating the origin and source of the infection [4, 8, 12, 19].

From these molecular approaches, RFLP method has been extensively validated and used for differentiation of* Fasciola* species [24, 25]. With help of three four-base-cutting endonucleases,* Hinf I*,* MspI,* and* RsaI* on COI gene, Hashimoto et al. showed different patterns of RFLP for fasciolids [26]. Marcilla et al. reported that digestion of 28S rDNA by the restriction enzyme,* AvaII* or* DraII*, can accurately distinguish species of* Fasciola* [27].

RFLP method has been used for distinguishing of* Fasciola* species in Iran in a number of studies, using* DraI* and* BfrI* for 18s DNA region,* TasI* for ITS1 region,* AvaII* and* DraII* for 28s DNA, and* BamH1* and* PagI* for ITS2 [13–16].

In our study, using double digestion with two restriction enzymes,* MspI* and* KpnI*, different patterns were observed for two* Fasciola* species. RFLP patterns of ITS1 and ITS2 genes showed that both* F. gigantica* and* F. hepatica* are sympatric in this region while no hybrid forms were detected in the region.

Findings of our study, regarding the genotypes of ITS1 region, demonstrated six variable nucleotides sites between two species of* Fasciola*. This is consistent with most of previous studies [6, 19, 28]. Moreover, genotype analysis of ITS2 region revealed seven variable nucleotides sites between the two species and this again is in line with previous studies [22, 29]. However, differences at six nucleotide sites of ITS2 have been reported in few studies [30, 31]. Phylogenic tree of ITS1 and ITS2 showed that flukes are scattered as pure* F. hepatica* and* F. gigantica* clades, suggesting that two genotypes of* Fasciola* are able to infect animals and probably human in southwest of Iran. The phylogenetic trees showed a close relationship of Iranian isolates with those isolates from different regions of the world. Comparison of ITS sequences of* Fasciola* isolates of different hosts from different countries indicates that a high species-specific homogeneity exists in each region. ITS2 sequence splits the* F. gigantica* population into those from Africa and those from Asia. This should be interpreted in conjunction with recent finding which suggests that Asian and African* F. gigantica* may be separate species [32]. Substantial genetic divergence between morphologically indistinguishable populations of* Fasciola* suggests the possibility of cryptic speciation [32].

Mitochondrial markers can exhibit a high level of intraspecific diversity in* F. hepatica* within a relatively confined geographic region [33]. Based on these markers, flukes of our study were clustered in two phylogenetically distinguishable clades. This finding is coinciding with results of similar related studies in Iran and also other Asian and African countries [4, 6, 12, 19]. Indeed, mitochondrial, rather than nuclear, sequences strongly support this notion and suggest that the flukes characterized in this study are related to the Asian* F. gigantica*.

In conclusion, the present study demonstrated that the liver fluke isolated from three main hosts' species of* Fasciola* in the southwest of Iran represented the two species of* F. hepatica* and* F. gigantica*.

The current study explored the molecular characteristics of* Fasciola* in the area and more studies are needed to determine the other aspect of fascioliasis in this new emerging focus of human fascioliasis in Iran.

## Figures and Tables

**Table 1:** The name and sequences of the primers for PCR.

Gene	Name	Sequence	Size of PCR product (bp)
COI	Ita 8	5′-ACGTTGGATCATAAGCGTGT-3′	438
	Ita 9	5′-CCTCATCCAACATAACCTCT-3′	
NDI	Ita 10	5′-AAGGATGTTGCTTTGTCGTGG-3′	535
	Ita 2	5′-GGAGTACGGTTACATTCACA-3′	
ITS1	ITS1-F	5′-TTGCGCTGATTACGTCCCTG-3′	600
	ITS1-R	5′-TTGGCTGCGCTCTTCATCGAC-3′	
ITS2	ITS2-F	5′-TGTGTCGATGAAGAGCGCAG-3′	505
	ITS2-R	5′-TGGTTAGTTTCTTTTCCTCCGC-3′	

**Table 2:** Comparative morphometrical data on adults *F. hepatica* and *F. gigantica* from naturally infected cattle, sheep, and goat of the Kohgiluyeh and Boyer-Ahmad province, southwest of Iran.

Parameters (mm)	*F. hepatica *			*F. gigantica *
Host	Cattle	Sheep	Goat	Cattle
Number of adult flukes	19	19	22	18
Body length (BL)	15.09–36.84*	19.77–37.37	20.58–32.58	27.22–51.19
	23.08 ± 5.27**	28.17 ± 5.14	27.31 ± 3.67	41.08 ± 6.12

Maximum body width (BW)	6.23–13.22	8.37–13.88	9.07–21.41	7.03–8.98
	9.93 ± 1.79	11.33 ± 1.47	11.89 ± 2.49	8.14 ± 0.62

Body width at ovary level (BWOv)	2.6–5.26	3.36–6.01	3.95–10.39	2.76–4.3
	3.95 ± 0.68	4.7 ± 0.84	5.45 ± 1.31	3.36 ± 0.49

Cone length (CL)	1.70–2.88	1.77–3.03	1.62–3.13	2.54–3.72
	2.26 ± 0.36	2.47 ± 0.43	2.26 ± 0.38	3.1 ± 0.35

Cone width (CW)	2.08–3.94	2.3–4.38	1.89–3.69	3.01–4.34
	2.92 ± 0.41	3.13 ± 0.62	2.91 ± 0.41	3.56 ± 0.34

Maximum diameter of oral sucker (OS max)	0.49–0.96	0.33–0.88	0.57–0.95	0.61–1.05
	0.69 ± 0.14	0.7 ± 0.12	0.71 ± 0.1	0.81 ± 0.11

Minimum diameter of oral sucker (OS min)	0.57–1.13	0.51–1.23	0.65–1.13	0.84–1.13
	0.78 ± 0.13	0.85 ± 0.17	0.83 ± 0.12	0.96 ± 0.08

Maximum diameter of ventral sucker (VS max)	0.84–1.28	0.91–1.76	0.64–1.62	1.31–2.09
	1.1 ± 0.11	1.29 ± 0.20	1.21 ± 0.19	1.76 ± 0.2

Minimum diameter of ventral sucker (VS min)	0.84–1.52	0.82–1.77	0.98–1.38	1.21–4.1
	1.06 ± 0.15	1.19 ± 0.19	1.17 ± 0.11	2.99 ± 0.74

Distance between anterior end of body and ventral sucker (A-VS)	1.58–3.3	1.84–3.36	0.96–3.52	2.18–2.93
	2.43 ± 0.45	2.57 ± 0.48	2.45 ± 0.53	2.59 ± 0.24

Distance between suckers (OS-VS)	1.01–2.31	1.25–3.04	1.38–2.53	1.34–2.28
	1.73 ± 0.38	1.97 ± 0.48	1.81 ± 0.31	1.79 ± 0.24

Distance between ventral sucker and union of vitelline glands (VS-Vit)	9.22–20.93	8.58–20.93	11.24–19.64	11.4–27.99
	12.47 ± 3.27	16.06 ± 3.3	15.93 ± 2.24	19.79 ± 6.49

Distance between Vit and posterior end of body (Vit-P)	2.79–12.23	6.89–13.41	3.02–11.96	8.39–26.71
	7.16 ± 2.6	8.83 ± 1.85	8.13 ± 2.31	17.83 ± 4.91

Distance between VS and posterior end of body (VS-P)	12.38–32.29	17.01–32.74	17.36–29.14	22.38–46.93
	12.74 ± 5.21	24.89 ± 4.62	23.79 ± 3.3	37.67 ± 6.39

Pharynx length (PhL)	0.46–1.15	0.65–1.26	0.81–1.38	0.67–1.09
	0.86 ± 0.18	0.99 ± 0.18	0.99 ± 0.13	0.91 ± 0.11

Pharynx width (PhW)	0.32–0.69	0.32–0.71	0.31–1.05	0.37–1.22
	0.44 ± 0.1	0.47 ± 0.1	0.52 ± 0.17	0.54 ± 0.17

Testicular space length (TL)	7.31–18.82	7.03–19.11	9.45–18.34	3.52–24.41
	10.66 ± 2.99	13.9 ± 3.04	13.55 ± 2.24	16.45 ± 5.37

Testicular space width (TW)	4.1–8.81	5–9.59	4.03–8.75	3.24–5.2
	6.09 ± 1.08	7.1 ± 1.19	7.16 ± 1.09	4.44 ± 0.48

Body area (BA)	94.01–398.97	165.47–478.81	212.23–458.38	234.9–452.51
	235.2 ± 86.6	322.51 ± 83.96	322.55 ± 63.36	334.34 ± 56.58

Oral sucker area (OSA)	0.3–1.08	0.16–1.07	0.4–0.92	0.55–1.18
	0.55 ± 0.19	0.6 ± 0.21	0.59 ± 0.16	0.78 ± 0.15

Ventral sucker area (VSA)	0.7–1.76	0.79–3.11	0.62–2.2	1.21–4.1
	1.17 ± 0.23	1.57 ± 0.5	1.43 ± 0.33	2.99 ± 0.74

Pharynx area (PhA)	0.16–0.74	0.25–0.84	0.29–1.44	0.38–0.81
	0.39 ± 0.13	0.47 ± 0.17	0.53 ± 0.26	0.49 ± 0.11

BL/BW ratio (BL/BW)	1.83–3.4	1.15–3.44	1.82–3.43	3.15–6.09
	2.32 ± 0.33	2.43 ± 0.49	2.37 ± 0.43	5.06 ± 0.86

Ratio between sucker areas (OSA/VSA)	0.28–0.89	0.18–0.7	0.24–0.77	0.13 ± 0.6
	0.47 ± 0.17	0.4 ± 0.14	0.43 ± 0.14	0.27 ± 0.09

Ratio between BWOv and CW (BWOv/CW)	0.32–1.85	1.02–1.86	0.44–2.17	0.78–1.11
	1.29 ± 0.3	1.51 ± 0.24	1.73 ± 0.53	0.93 ± 0.09

Ratio between BL and VS-P (BL/VS-P)	1.06–1.26	0.89–1.19	0.11–1.18	1.05–1.21
	1.17 ± 0.48	1.12 ± 0.04	1.09 ± 0.22	1.09 ± 0.05

*Minimum–maximum (mm).

**Mean ± SD.
